# Silicon Enhances Plant Vegetative Growth and Soil Water Retention of Soybean (*Glycine max*) Plants under Water-Limiting Conditions

**DOI:** 10.3390/plants11131687

**Published:** 2022-06-25

**Authors:** Saroj Kumar Sah, Kambham Raja Reddy, Jiaxu Li

**Affiliations:** 1 Department of Biochemistry, Molecular Biology, Entomology and Plant Pathology, Mississippi State University, Mississippi State, MS 39762, USA; saroj1021@gmail.com; 2 Department of Plant and Soil Sciences, Mississippi State University, Mississippi State, MS 39762, USA; krreddy@pss.msstate.edu

**Keywords:** abiotic stress, water deficit, vegetative growth, root volume, water retention

## Abstract

Silicon has been implicated as a factor affecting the degree of resistance to abiotic stresses in several plant species. However, the role of silicon in soybean (*Glycine max*) under water-limiting conditions is not yet fully understood. This study was conducted to evaluate the effects of silicon application on the vegetative growth of two soybean cultivars (Asgrow 5332 and Progeny 5333) grown under water-limiting conditions. Silicon was provided by adding silicate to the soil. Water-limiting treatments were imposed on plants at two vegetative growth stages for 20 days by irrigating with a reduced amount of water (66% or 33% of the required water). Silicate application enhanced plant height, leaf area, and total dry weight of soybean plants. Significant increases in root volumes were observed in both the silicate-treated cultivars compared to the control plants under water-limiting conditions (33% irrigation). Net photosynthesis and stomatal conductance were decreased, but the quantum efficiency of photosystem II (Fv’/Fm’) did not change under the same irrigation condition, which indicates photosynthesis downregulation through stomatal limitation. Silicate-treated plants in both cultivars had higher water use efficiency as compared to control plants under water-limiting conditions (irrigated with 66% or 33% of required water). Under water-limiting conditions, the soil moisture content was significantly higher in pots containing silicate than in those without added silicate, suggesting that silicon application improves water holding capacity. Taken together, the results from this study indicate that silicon application can improve the vegetative growth of soybeans under low water conditions by increasing the water use efficiency of plants and enhancing the soil’s ability to retain moisture.

## 1. Introduction

In the coming years, the growing scarcity of water accessible for irrigation triggered due to urbanization and depletion of aquifers poses severe threats to sustainable crop production. Various abiotic stresses strongly affect agricultural productivity, among which drought is the primary environmental constraint limiting plant growth and yield. A study conducted between 1981 to 2010 reported that fluctuation in temperature and rainfall accounted for almost 70% of the variation seen in the United States crop productivity [1]. It also predicted that, between 2010 and 2040, productivity could drop by 2.8% and 4.3% a year, respectively. The top contributor to this decline was hotter summers in the corn (*Zea mays* L.) and soybean (*Glycine max*) growing regions of the Midwest of the United States [1]. 

Soybean is one of the most important crops worldwide, which provides a complete protein as it contains all essential amino acids for human beings [2,3]. It is rich in oil, 95% is used up as edible oil, and the remaining is used as fatty acids, soaps, and biodiesel as industrial products [3]. The US exports 1940 million bushels per year which is 49% of production and plays a significant role in the country’s economy [4]. However, future soybean production remains a challenging task due to changes projected in climate. Therefore, there is a great need to develop production systems to maintain consistent yields under water-limiting conditions. 

Silicon has recently been recognized as an essential element in plant nutrition and is considered to abate plant stresses, including drought [5]. Although silicon is most abundant, most of the sources of silicon are water-insoluble and not available directly to plants [6,7]. Several elements impact soil silicon availability to plants, such as soil type, temperature, organic matter, soil pH, and texture [8,9]. The concentrations of silicic acid in soil solutions range from 0.1 to 0.6 mM [10]. Plants take silicon via passive uptake and transport from the roots to the shoots in the form of monosilicic acid and deposit it as solid, amorphous, hydrated plant silica [11]. Once deposited, silicon is not remobilized. Silicon is transported in the plant via xylem using apoplastic transport and remains unpolymerized during this passage [10,12]. Silicon influences water relations under drought-stressed plants, which induces the formation of the silica cuticle double layer under the leaf epidermis that reduces water losses through cuticular transpiration [13]. Silicon also decreases stomatal conductance, which leads to turgor loss of guard cells associated with silicon deposition and modified cell wall properties [14]. Silicon is gaining more attention from plant biologists because of its reported dynamic roles in alleviating abiotic and biotic stresses [15]. Plants with silicon supplies generate stronger cell walls and show increased biomass production. Studies showed that silicon’s application improves plants’ growth and development in abiotic stress such as drought, salt, and heavy metal toxicity [16].

The understanding of the role of silicon in abiotic stress resistance is comparatively limited. However, important avenues of research in abiotic stress contexts are emerging [16,17,18]. However, the role of silicon in plant biology is poorly understood within water-limited production systems. Therefore, the primary objective of this study was to investigate the role of silicon in explicating the stress-response mechanisms of soybean in water-limiting conditions.

## 2. Results

### 2.1. Silicon Application on Vegetative Growth of Soybeans

**Figure 1:**
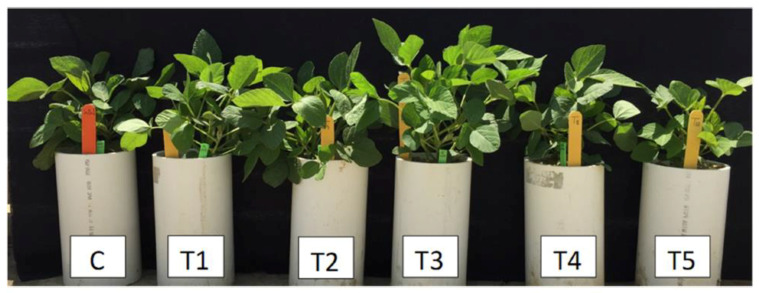
The effect of silicon on vegetative growth of soybean plants. Potassium silicate was incorporated into soils to give different silicon concentrations: T1 (50 ppm), T2 (150 ppm), T3 (500 ppm), T4 (1500 ppm), and T5 (2000 ppm). Asgrow 5332 soybean plants were grown in pots with soil and nutrient supply at natural light and temperature conditions for 45 days. The control pots (C) contained soils with added potassium chloride, which was used as a control to cancel the effect of potassium.

The first set of experiments conducted was to determine the optimal concentration of silicon on soybean growth. As shown in Figure 1, 500 ppm of silicon was the optimal concentration for improving soybean vegetative growth.

### 2.2. Effects of Silicon on Vegetative Growth of Soybeans (7–27 Days after Emergence) Grown under Water-Limiting Conditions

**Figure 2:**
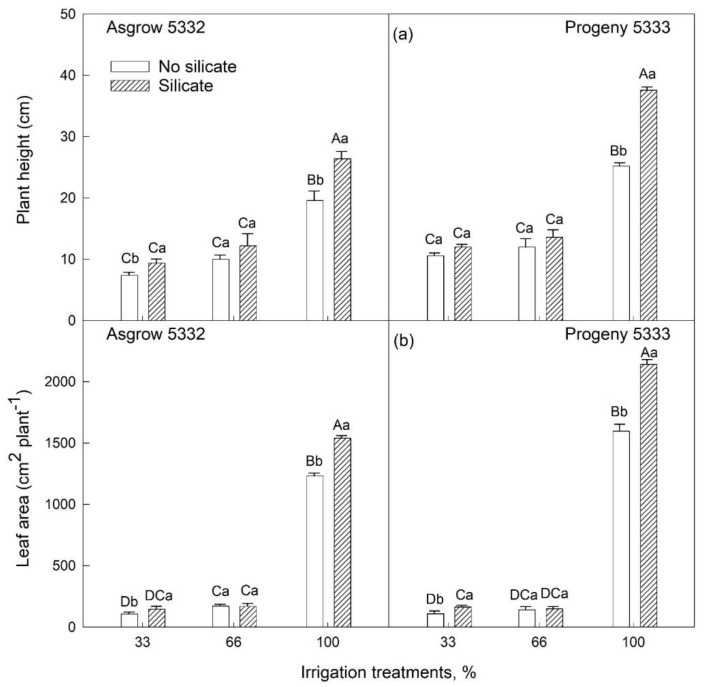
The influence of silicate application on soybean (**a**) plant height and (**b**) whole-plant leaf area across three irrigation treatments, measured at 27 days after emergence of the seedling. Three irrigation treatments (33%, 66%, or 100% of required water) were imposed on plants for 20 days. The values are mean and standard errors of five replications in each treatment. The error bars correspond to standard error. The bars followed by the same letters (lower-case letters within silicate and no silicate and upper-case letters within irrigation and silicate treatments) did not differ significantly at 5% by Tukey’s test.

In cultivar Asgrow 5332, there was a difference in plant height at 33% irrigation treatment. However, no significant difference was observed in another cultivar, Progeny 5333, at 27 days of emergence in silicate treatment as compared to non-silicate-treated plants. Plant height in 100% irrigation treatments showed a significant difference at 27 days after emergence (Figure 2a), indicating that the Si application had increased the growth rate as compared to control treatments. The growth rate in 100% irrigation treatment was higher in silicate-treated plants. The interactive effects of silicate treatment and cultivar for plant height account for the significant difference between silicate-treated plants of cultivar Progeny 5333, i.e., 37.6 cm, and Asgrow 5332, which was 26.4 cm at 100% irrigation treatment with silicate (Figure 2a).

Furthermore, higher leaf area was observed in silicate-treated plants as compared to plants grown under standard conditions with 100% irrigation. There was no statistical difference in 66% of irrigation treatments in both cultivars at 27 days of emergence. In contrast, in 33% of irrigation treatments, silicate-treated plants had higher leaf area than non-silicate-treated plants in both cultivars. In 100% irrigation treatments, leaf area was higher in silicon-treated plants than control plants (Figure 2b).

**Figure 3:**
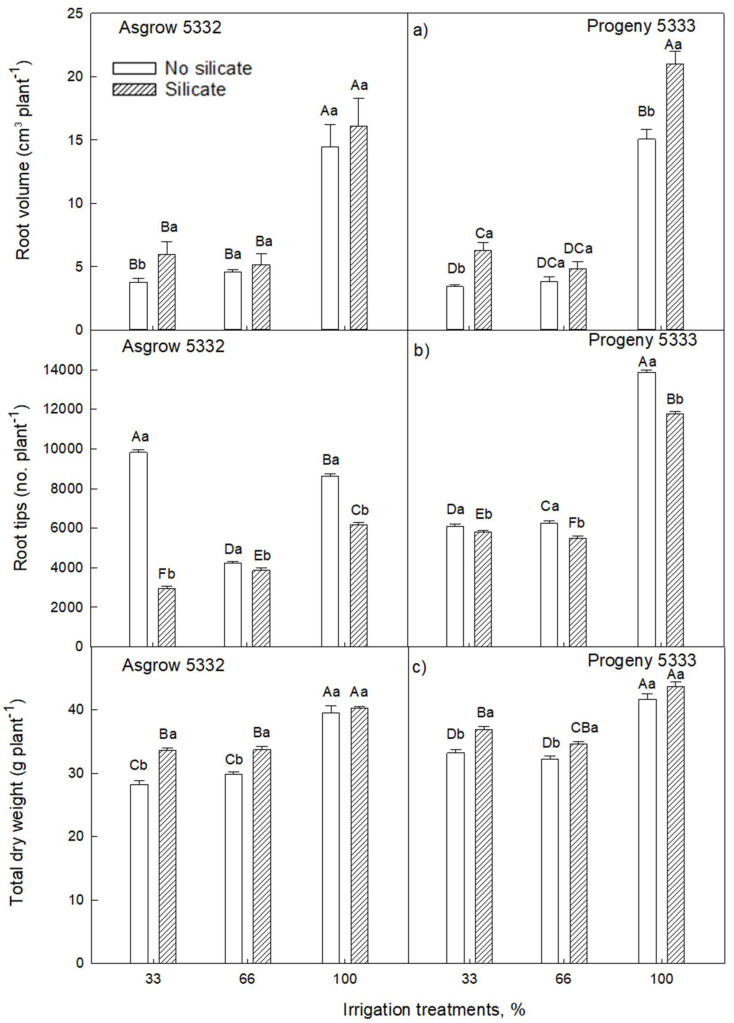
The influence of silicate application on soybean (**a**) root volume, (**b**) root tips, and (**c**) plant total dry weight of three irrigation treatments, measured at 27 days after emergence of the seedling. Three irrigation treatments (33%, 66%, or 100% of required water) were imposed on plants for 20 days. The values are mean and standard errors of five replications in each treatment. The error bars correspond to standard error. The bars followed by the same letters (lower-case letters within silicate and no silicate and upper-case letters within irrigation and silicate treatments) did not differ significantly at 5% by Tukey’s test.

There was a significant difference in root volume among silicate treatments and irrigation treatments (Figure 3a). With 100% irrigation, root volumes were increased in silicate-treated plants compared to the control plants in cultivar Progeny 5333. Significant increases in root volumes were observed at 33% irrigation treatment in both the cultivars in silicate-treated plants compared to the control plants (Figure 3a). However, root volume did not differ significantly in 66% irrigation in both the cultivars compared to control plants. In both the cultivars, there was a substantial reduction in root tips in silicate-treated plants compared to the control plants across all the irrigation treatments (Figure 3b). There was approximately more than 10% percent increase in silicate-treated plants in 33% and 66% irrigation treatments, respectively, compared to control plants in cultivar Asgrow 5332. Similarly, there was a more than 7% percent increase in silicate-treated plants in 33% and 66% irrigation treatments, respectively, as compared to control plants in cultivar Progeny 5333 (Figure 3c). The total dry weights significantly increased in silicate-treated plants under water-limiting conditions (irrigated with 33% or 66% of required water) (Figure 3c).

Significant differences were observed among water-limiting treatments and silicate treatments for the measured parameters, such as plant height, node number, leaf area, stem dry weight, root dry weight, root-shoot ratio, total dry weight, root surface area, root length, root diameter, root volume, root tips, and root crossing (Table 1). There was no interaction in node number among cultivars, whereas there was interaction among silicate treatments (Table 1).

### 2.3. Effects of Silicon on Growth and Physiology of Soybeans (25–45 Days after Emergence) Grown under Water-Limiting Conditions

**Figure 4:**
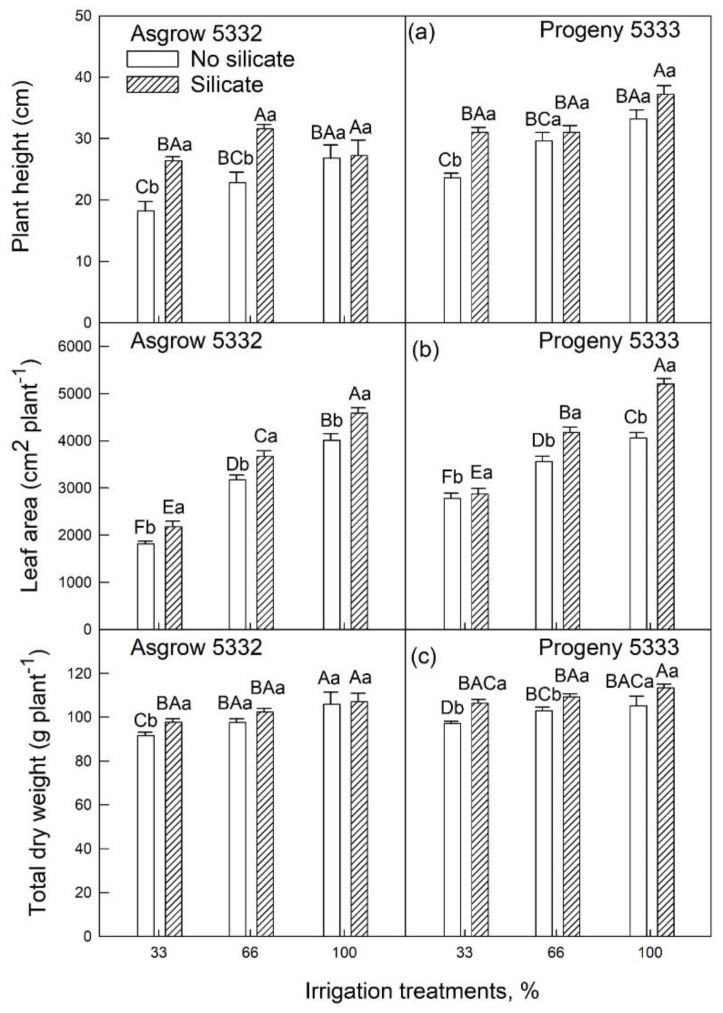
The influence of silicate application on two soybean cultivars (**a**) plant height, (**b**) whole-plant leaf area, and (**c**) total dry weight across three irrigation treatments, measured at 45 days after emergence of the seedling. Three irrigation treatments (33%, 66%, or 100% of required water) were imposed on plants for 20 days. The values are mean and standard errors of five replications in each treatment. The error bars correspond to standard error. The bars followed by the same letters (lower-case letters within silicate and no silicate and upper-case letters within irrigation and silicate treatments) did not differ significantly at 5% by Tukey’s test.

Plant height was increased in silicate-treated plants by 31% and 27% in 33% and 66% irrigation treatment, respectively, compared to control plants in cultivar Asgrow 5332 (Figure 4a). Similarly, the plant height was increased in Si-treated plants by 23% in 33% irrigation treatment. In contrast, as compared to control plants, no significant differences were observed in 66% and 100% irrigation treatment in cultivar Progeny 5333 (Figure 4a). There was a sharp increase in the leaf area in silicate-treated plants compared to plants grown without silicate treatment. In both cultivars, leaf area decreases as the level of water deficit increases. The total leaf area was larger in silicate-treated plants than in plants without silicate treatment under each irrigation condition (100%, 66%, or 33%) (Figure 4b). Compared to the control, there was a significant increase in total dry weight in silicate-treated plants (Figure 4c). In cultivar Asgrow 5332 compared to control, the total dry weight was increased by approximately 7% in silicate-treated plants in 33% irrigation treatments. Likewise, in cultivar Progeny 5333, the total dry weight was increased by 8% in 33% irrigation treatments compared to control plants. There was no remarkable difference in root dry weight, longest root length, and root volume in either cultivar or irrigation treatments (Table 2).

**Figure 5:**
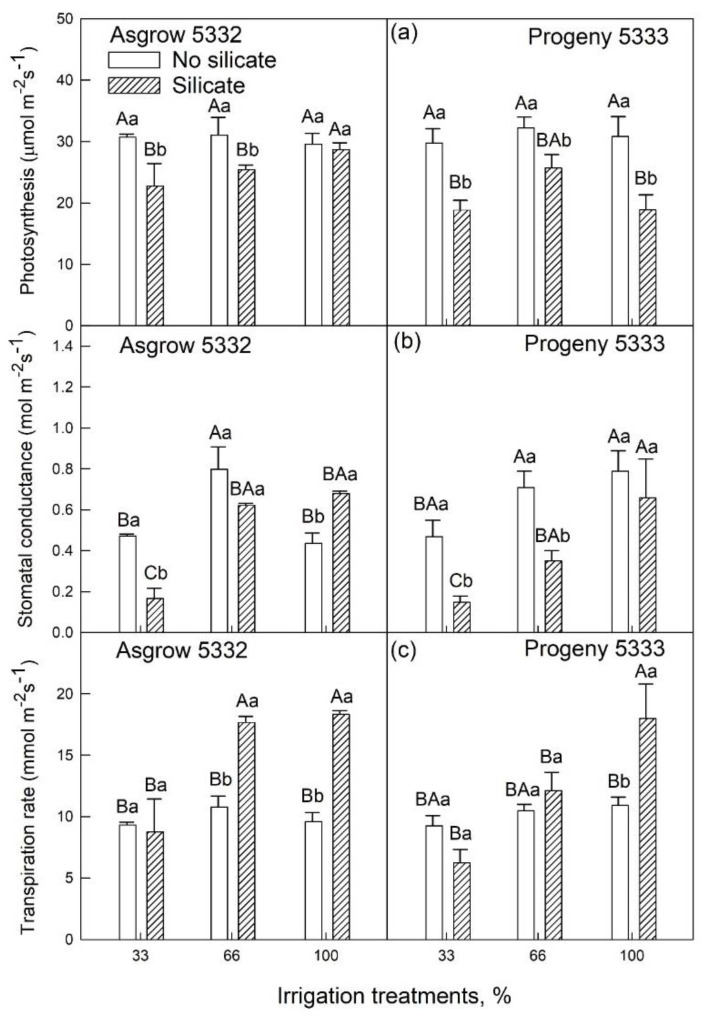
The influence of silicate application on two soybean cultivars’ (**a**) photosynthesis, (**b**) stomatal conductance, and (**c**) transpiration rate across three irrigation treatments, measured at 45 days after emergence of the seedling. The error bars correspond to standard error. The bars followed by the same letters (lower case letters within silicate and no silicate and upper-case letters within irrigation and silicate treatments) did not differ significantly at 5% by Tukey’s test.

**Figure 6:**
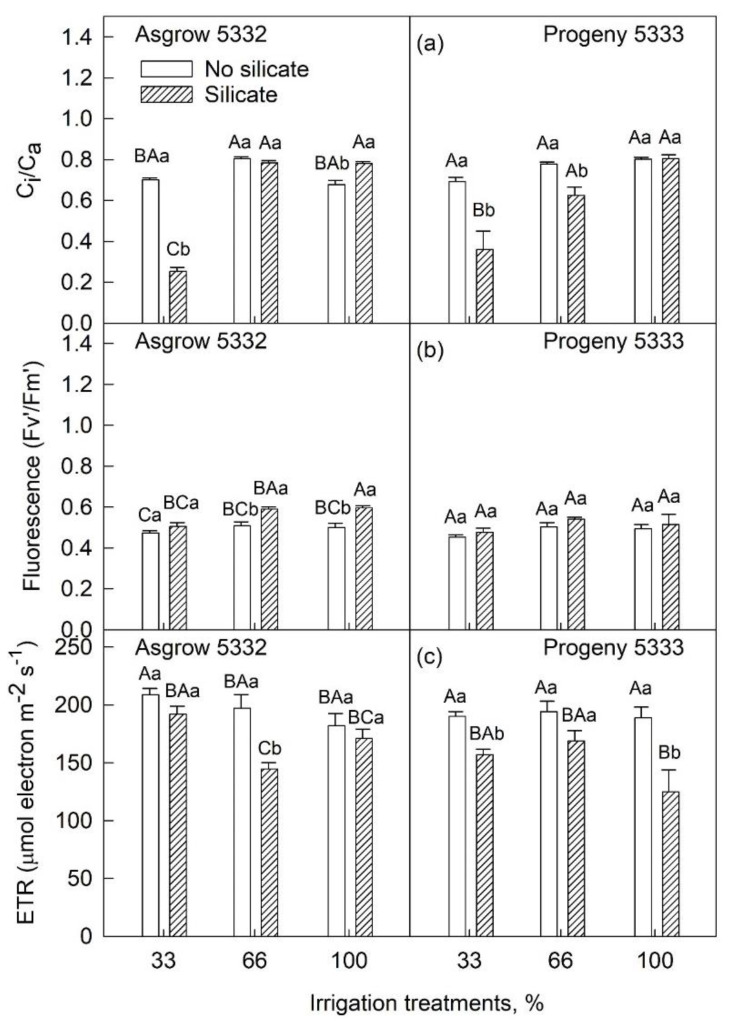
The influence of silicate application on two soybean cultivars: (**a**) the ratio of internal to external carbon dioxide concentration (C_i_/C_a_), (**b**) fluorescence (Fv’/Fm’), and (**c**) electron transport rate (ETR) across three irrigation treatments, measured at 45 days after emergence of the seedling. The values are mean and standard errors of five replications in each treatment. The error bars correspond to standard error. The bars followed by the same letters (lower-case letters within silicate and no silicate and upper-case letters within irrigation and silicate treatments) did not differ significantly at 5% by Tukey’s test.

All the measured physiological parameters such as photosynthesis, stomatal conductance, the ratio of internal and external CO_2_ concentration, quantum efficiency, electron transport rate, and water use efficiency showed significant differences in silicate-treated plants as compared to plants without silicate treatments (Table 2). The net photosynthesis rates in soybean leaves with silicate treatments were lower than in soybean leaves without silicate treatments under each irrigation condition (100%, 66%, or 33%) (Figure 5a) except Asgrow 5332 at 100% irrigation treatment. Under water-limiting conditions (66% or 33%), stomatal conductance decreased more in silicate-treated plants than in control plants in Progeny 5333. In contrast, in Asgrow 5332, only 33% of irrigation treatment stomatal conductance was reduced (Figure 5b). In cultivar Asgrow 5332, the transpiration rate was higher in silicate-treated plants than in control plants under 66% and 100% irrigation. Likewise, in Progeny 5333, the transpiration rate was higher in silicate-treated plants under 100% irrigation, but no significant differences were found in 33% and 66% irrigation treatments (Figure 5c). There was a significant reduction in the proportion of C_i_/C_a_ in silicate-treated plants in 33% of irrigation treatments in both the cultivars compared to control plants (Figure 6a). There was no significant difference observed in 66% of irrigation treatments in cultivar Asgrow 5332, whereas there was a substantial amount of reduction of C_i_/C_a_ observed in cultivar Progeny 5333 (Figure 6a). 

**Figure 7:**
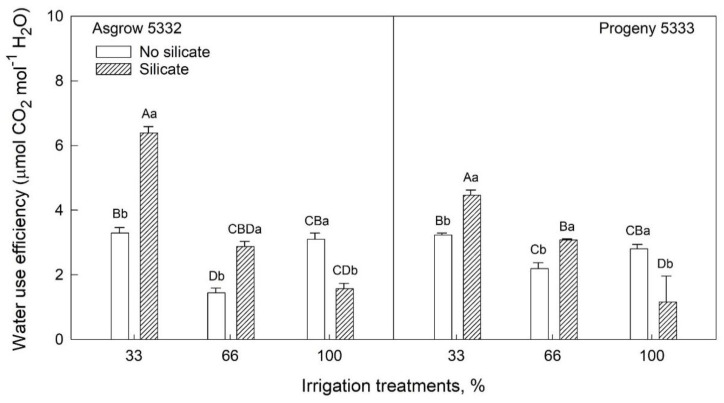
The influence of silicate application on two soybean cultivars’ water-use efficiency across three irrigation treatments, measured at 45 days after emergence of the seedling. The values are mean and standard errors of five replications in each treatment. The error bars correspond to standard error. The bars followed by the same letters (lower-case letters within silicate and no silicate and upper-case letters within irrigation and silicate treatments) did not differ significantly at 5% by Tukey’s test.

Chlorophyll fluorescence parameters can provide helpful information about the photosystem II activity of plants. In cultivar, Asgrow 5332, the quantum efficiency of PSII (Fv’/Fm’) was significantly higher in silicate-treated plants than in plants without silicate treatments under 66% and 100% irrigation (Figure 6b). No significant difference was observed in Fv’/Fm’ in cultivar Progeny 5333 with all irrigation treatments (Figure 6b). The electron transport rate was significantly lower in silicate-treated Asgrow 5332 plants under 66% irrigation treatment, while in cultivar Progeny 5333 electron transport rate was lower in silicate-treated plants under 100% irrigation treatment (Figure 6c). Water use efficiency (WUE) is a critical parameter for the growth and development of plants. WUE is calculated as the ratio of biomass produced by soybean plants to actual total water use. As shown in Figure 7, silicate-treated plants in both cultivars had higher WUE than control plants under water-limiting conditions (irrigated with 66% or 33% of required water). However, under 100% irrigation treatment, WUE was lower in silicate-treated plants than in control plants in both cultivars. 

### 2.4. Silicon Application on Soil Moisture Content under Water-Limiting Conditions

**Figure 8:**
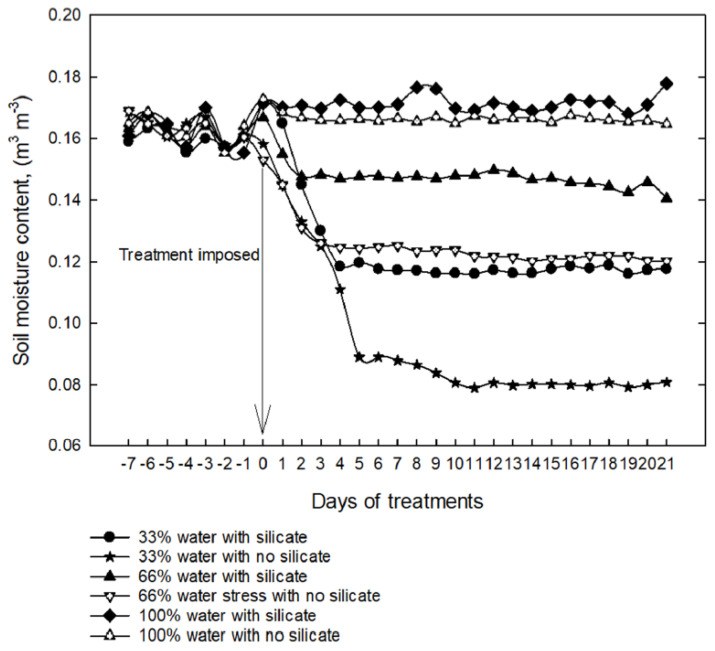
Volumetric soil moisture content in soil growing 7-day-old seedlings under water-limiting conditions. The arrow indicates the day on which water-limiting treatments were imposed.

**Figure 9:**
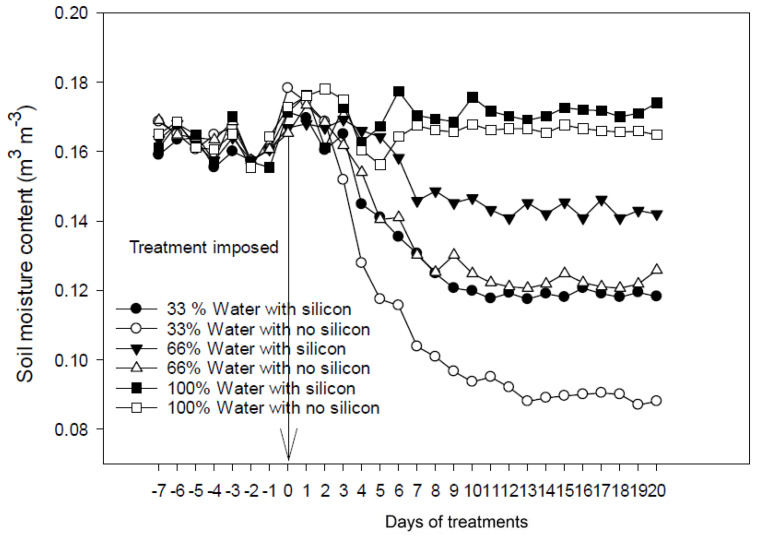
Volumetric soil moisture content in soil growing 25-day-old seedlings under water-limiting conditions. The arrow indicates the day on which water-limiting treatments were imposed.

During the first seven days, (−7 to −1) of measurements (Figure 8 and Figure 9), all plants growing in pots were irrigated with an average amount of water (100%). On day 0 and afterward, plants were supplied with a normal (100%) or reduced (66% or 33%) amount of water (Figure 8 and Figure 9). To impose water-limiting treatments on 7-day-old seedlings, the soil moisture content gradually decreased and reached a relatively constant lower level throughout the remaining experimental period (Figure 8). Under water-limiting conditions (66% or 33%), the soil moisture content was significantly higher in pots containing silicate than in those without added silicate (Figure 8). At 33% irrigation treatment with silicate application, the soil moisture content was as high as 66% irrigation with no silicate application (Figure 8). Similar results were obtained with the experiments imposing water-limiting treatments on 25-day-old seedlings; the soil moisture content was significantly higher in pots containing silicate than in those without added silicate under water-limiting conditions (Figure 9).

## 3. Discussion

In this study, experiments were conducted at early root and canopy developmental stages to identify the role of silicon in the growth of soybean plants under limited water conditions. Although some studies have reported the role of silicon in the growth and development of several plant species, including wheat [13], rice [19], sorghum [20,21], Kentucky bluegrass [22], and soybean [23], under drought conditions, no studies to date have reported the role of silicon in different levels of irrigation treatments. Therefore, this study is imperative in identifying the role of silicon in plant growth and physiology under water-limiting conditions. The outcome of the present study of increasing soybean growth by silicon under water-limited conditions has implications for managing the crop under rainfed and limited irrigation agricultural production systems. 

In the present study, we found that the addition of silicon resulted in an improvement in the growth and development of soybean plants. We conducted experiments at two vegetative growth stages of the soybean crop with multiple irrigation regimes. In this study, we used two cultivars: one is determinate (Progeny 5333), and the other is indeterminate (Asgrow 5332). The results showed that plant height, leaf area, and total dry weight differed in silicate-treated plants compared to plants grown without silicate treatment (Figure 2, Figure 3 and Figure 4). Thus, silicate application improved soybean plants’ vegetative growth and leaf biomass under both well-watered and water-limited conditions. These findings are comparable with observations in other crops, for example, studied in wheat, for which phosphorous application improved dry matter production in both wet and dry conditions [24]. Similar growth by silicon applications has been reported in other crops [20,21,22].

This study showed that silicate application influences root growth and development. Root volume significantly increased in silicate-treated plants compared to plants grown without silicate treatment in cultivar Progeny 5332 (Table 1, Figure 3a). In cultivar Asgrow 5332, there was a significant reduction of root tips, i.e., 8 to 78 percent decrease in silicate-treated plants as compared to control plants, whereas, in cultivar Progeny 5333, root tips were decreased by 5 to 15% in silicate-treated plants as compared to the control (Figure 3b). Besides the root tips, there is also the interaction of root surface area, root diameter, and the root-shoot ratio between silicate-treated plants as compared to controls along with irrigation treatments (Table 1). The study in maize showed that reduced lateral root branching density improves drought tolerance [25]. Likewise, another study also reported that lateral roots contribute to the silicon uptake in the rice plant, whereas root hairs do not [26]. Silicon also plays a role in alleviating root growth and development under a potassium-deficient medium [27].

Photosynthesis is one of the primary parameters affected by abiotic stress. Photosynthesis and stomatal conductance were reduced in silicate-treated plants compared to control plants under water-limiting conditions (33% and 66% irrigation). However, under normal water conditions (100% irrigation), stomatal conductance was increased (Figure 5). These findings were comparable with Saud and co-workers, who showed a strong relationship between photosynthesis with stomatal conductance and leaf green color, severely affected by drought stress [22]. The transpiration rate was lower under water-limiting conditions (33% irrigation, Figure 5c) in silicate-treated plants compared to plants grown without silicate treatment. Silicon is required to form the cuticle-silica double layer under the leaf epidermis, which could be the reason for the decreased transpiration rate in Si-treated plants compared to control plants [28]. Therefore, a decrease in silicon application transpiration is one of the mechanisms for a silicon-mediated increase in stress tolerance. Additionally, silica deposited in the tissues helps alleviate drought stress by reducing transpiration and improving light interception [6].

The quantum efficiency of photosystem II (Fv’/Fm’) did not change under the same irrigation regime (Figure 6b), which indicates photosynthesis downregulation may drive through stomatal limitation [29]. The Fv’/Fm’ trend increased along with increased irrigation in silicate-treated plants compared to the control plants in cultivar Asgrow 5332, whereas it was relatively constant in cultivar Progeny 5333. A different pattern was observed in an increase in the Fv’/Fm’ of rice plants subjected to drought stress with silicon application [19]. Silicon can induce photosynthesis-relevant genes in rice [30]. Silicon upregulated the level of PsbY, a low molecular mass subunit of the oxygen-evolving complex of PSII. Silicon stimulated the expression of PsaH, a gene necessary for the light-harvesting complex function. The expression of *PetC*, a gene encoding a Fe-S protein of cytochrome bf complex, was also upregulated by silicon treatment. Overall, silicon can upregulate photosynthetic machinery genes. 

Water use efficiency (WUE) is an essential parameter in agriculture, and improving the WUE of crops is becoming the main objective for agriculture and food security goals. In the present study, WUE was shown to increase silicate-treated plants under water-limiting conditions (33% and 66% irrigation) in both the cultivars, but WUE is decreased in 100% irrigation treatment in both the cultivars as compared to the respective control plants (Figure 7). These findings were comparable with observations in other crops [21,22]. The increase in WUE suggests that silicon application may serve as a valuable strategy for water management.

A key finding from this study is that silicate application could improve soil moisture retention under water-limiting conditions (Figure 8 and Figure 9). Similarly, the capacity of soil to retain water was enhanced with biochar application [31,32]. Another study reported that biochar application increased quinoa’s growth, drought tolerance, and water use efficiency [33]. Studies have suggested that improved water holding capacity and improved crop nutrient availability could be two main mechanisms for increasing the productivity of crops [34]. Our results indicate that silicon plays an essential role in modulating soil moisture contents by improving the water holding capacity of the plants, which could contribute to improved crop productivity under water-limiting conditions. The results obtained in this study support the above conclusion and reinforce the beneficial effects of silicon on the increase in plant height, leaf area, soil water retention, and biomass, hence on the improvement of physiological functionality leading to enhanced growth and development of plants.

In the present study, we showed that silicon application could enhance soybean growth through increments in plant height, leaf area development, and thus increased biomass. Improving early-season canopy development will improve yields under rainfed and water-limited agriculture production systems. Our study provides insight and is a step forward in establishing silicon’s role in enhancing soybean plants’ performance under water-limiting conditions. Currently, we are conducting experiments in the field to investigate the role of silicon in improving soybean yield in rainfed and water-limiting conditions.

## 4. Materials and Methods

### 4.1. Experimental Facility and Seed Material

The experiments were conducted in a natural environment at the Rodney Foil Plant Science Research Center, Mississippi State University, Mississippi State (33°28′ N, 88°47′ W), MS, USA. This study used two different cultivars, Asgrow 5332 (indeterminate) and Progeny 5333 (determinate). They were evaluated at three levels of soil moisture. The average temperature and relative humidity varied between 18–29 °C and 46–94%, respectively, during the experimental period. The average solar radiation was 505 langley/day, and the mean photoperiod was approximately 11 h [35]. The fungicide-treated seed was planted in polyvinyl chloride (PVC) pots (15.2 cm diameter and 30.2 cm height) in all experiments. Each pot was filled with a 5.5 kg mixture of soil medium consisting of sand and soil (3:1 ratio) with 500 g of gravel at the bottom. The pots were arranged in two rows, oriented in an east-to-west direction with 1-m spacing between rows. In each pot, four seeds were sown and, after five days of emergence, thinned to one plant in each pot till harvest.

### 4.2. Silicate and Soil Moisture Treatments

As the silicon source, potassium silicate soluble powder from AgSil^®^ 16H (PQ Corporation, Valley Forge, PA, USA) was used. Sixty polyvinyl chloride pots, five pots for each treatment, filled with a 5.5 kg mixture of sand and soil (3:1 ratio) with and without 500 ppm of silicon, were used in the study. Plants were irrigated through an automated and computer-controlled drip system, delivered three times a day at 08:00, 12:00, and 17:00 with Hoagland’s nutrient solution [36]. This study imposed three different irrigation treatments (33%, 66% and 100%). In 100% irrigation treatment, the water was supplied at full field capacity, whereas in 33% irrigation treatment, only 33% of water was provided, and in 66% irrigation treatment, only 66% water was supplied. The water amount was calculated based on-field capacity water. Three soil moisture treatments were imposed on soybean seedlings (7 days of emergence) and continued for 20 days in the first set of experiments. In the second set of experiments, soil moisture treatments were imposed after 25 days of emergence and continued for 45 days of planting (Supplementary Figure S1). Treatment soil moisture levels were maintained based on daily measurements of soil moisture content. On rainy days, the pots were covered with plastic mounted on wooden frames to prevent rainwater from entering the pots (Supplementary Figure S2).

### 4.3. Seedling Growth and Biomass Yield

Plant height and node numbers were measured/counted on all plants. Stem lengths were estimated as the distance between the cotyledonary node and the main stem apex. LI-3100 (LI-COR, Inc., Lincoln, NE, USA) leaf area meter was used to measure the leaf area. Plant total dry weights, including leaves, stems, and roots, were recorded after oven drying for three days at 80 °C. The measurements were taken on five different replicate plants in each treatment.

### 4.4. Root Growth Parameters

The winRHIZO optical scanner (Regent Instruments, Inc., Québec, Canada) was used to scan the roots before drying the roots in the first set of experiments. After separating the stem from individual root systems of each plant, roots were washed by placing the pot on sieves and gently spraying with water. Roots images were acquired and analyzed for different parameters of roots using WinRHIZO Pro software (Regent Instruments, Inc.) as described by [35,36]. The measurements were taken on five different replicate plants in each treatment.

### 4.5. Photosynthesis Rate, Stomatal Conductance, Transpiration Rate, and Chlorophyll Fluorescence

The leaf net photosynthesis rate (Pn), stomatal conductance (gs), transpiration rate (Trans), electron transport rate (ETR), and chlorophyll fluorescence (Fv’/Fm’) were measured using a portable photosynthesis system (Li-6400; Li-COR Inc., Lincoln, NE, USA) with an integrated fluorescence chamber head (Li-COR 6400 leaf chamber fluorometer). These measurements were made on recently fully expanded leaves, third from the top, between 10:00 am and 1:00 pm. The leaf cuvette temperature was set to 30 °C, and carbon dioxide (CO_2_) concentration was set to 400 µmol m^−2^ s^−1^. The atmospheric CO_2_ concentration was controlled with a CO_2_ injection system controlled by the Li-6400. The measurements were taken on five different replicate plants in each treatment. The photosynthetic water-use efficiency of the plants was calculated as Pn/gs at an ambient CO_2_ concentration (C_a_) of 400 µmol mol^−1^, and the ratio of internal/external CO_2_ concentration (C_i_/C_a_) recorded by the instrument was measured [37].

### 4.6. Soil Moisture Content

Soil moisture data were collected using a soil moisture sensor device (HH2 moisture meter with ML2X- theta probe, Delta-T Devices, Burwell, Cambridge, UK) every day between 12:00 to 14:00 before and after imposing the treatments. Soil moisture content was monitored using a soil moisture meter (HH2 moisture meter with ML2X-theta probe, Delta-T device) on a daily basis throughout the experiments

### 4.7. Statistical Analysis

Data were subjected to analysis of variance with a split-plot design considering varieties and treatment as a source of variance. All the measurements in each treatment were used as replicates for testing the significance of treatments, and standard errors of the mean are provided in the tables and figures. The data were analyzed with the PROC GLM general linear model in SAS v.9.3 and Tukey’s Honestly Significant Difference (HSD) tested at a *p* = 0.05 probability level (SAS Institute, 2011) and were employed to distinguish differences among treatments for the measured parameters in each experiment. Multi-factorial analysis was performed using FACTORS: Cul, Ws Trt, Si Trt, and their interaction. Graphical analysis was performed with Sigma Plot 13.0 (Systat Software Inc., San Jose, CA, USA).

## Figures and Tables

**Table 1:** Analysis of variance across the silicate treatments and their interactions of growth and development, biomass production, and partitioning and physiological traits of soybean plants: plant height (PH), number of nodes (NN), leaf area (LA), leaf dry weight (LDW), stem dry weight (SDW), root dry weight (RDW), shoot dry weight (SHDW), root/shoot ratio (RS), total dry weight (TDW), root length (RL), root surface area (RSA), root diameter (RD), root volume (RV), root tips (RT), root forks (RF), root crossing (RC), canopy temperature (CT), and chlorophyll content (SPAD).

Source of Variation	PH (cm)	NN (no.)	LA (cm^2^)	LDW (g)	SDW (g)	RDW (g)	SHDW (g)	RS	TDW (g)	RL (cm)	RSA (cm^2^)	RD (mm)	RV (cm^3^)	RT (no.)	RF (no.)	RC (no.)	CT (°C)	SPAD
**Cultivar**	***	NS	**	NS	**	*	*	NS	**	NS	NS	NS	NS	***	NS	NS	NS	NS
**WS Trt**	***	***	***	***	***	***	***	***	***	***	***	***	***	***	***	***	**	**
**Si Trt**	***	**	**	NS	**	*	NS	*	**	*	*	***	***	***	NS	**	NS	NS
**Cul X WS Trt**	***	NS	***	**	**	**	**	***	*	*	NS	NS	*	**	NS	NS	NS	NS
**Cul X Si Trt**	NS	NS	NS	NS	NS	NS	NS	*	NS	NS	NS	*	NS	NS	NS	NS	NS	*
**WS Trt X Si Trt**	***	NS	**	NS	*	NS	NS	***	NS	*	NS	NS	NS	***	NS	***	NS	NS
**Cul X WS Trt X Si Trt**	NS	NS	NS	NS	NS	NS	NS	**	NS	NS	NS	NS	NS	NS	NS	NS	*	NS

The significance levels ***, **, *, and NS represent *p* ≤ 0.001, *p* ≤ 0.01, *p* ≤ 0.05, and *p* > 0.05, respectively. NS: non-significant Cul: cultivar, WS Trt: water stress (limiting) treatment, Si Trt: silicate treatment. The observations were recorded at 27 days after emergence of seedling.

**Table 2:** Analysis of variance across the silicate treatments and their interactions of growth and development, biomass production, and partitioning and physiological traits of soybean plants: plant height (PH), number of nodes (NN) and leaf area (LA), leaf dry weight (LDW), stem dry weight (SDW), root dry weight (RDW), shoot dry weight (SHDW), root/shoot ratio (RS), total dry weight (TDW), root length (RL), root branch number (RBN), and root volume (RV), canopy temperature (CT), net photosynthesis (Pnet), stomatal conductance (Cond), internal CO_2_ (C_i_), the ratio of internal/external CO_2_ concentration, quantum efficiency (Fv’/Fm’), electron transport rate (ETR), transpiration rate (Trans), and water use efficiency (WUE).

Growth and Developmental Traits												
Source of Variation	PH (cm)	NN (no.)	LA (cm^2^)	LDW (g)	SDW (g)	RDW (g)	SHDW (g)	RS	TDW (g)	RL (cm)	RBN (no.)	RV (cm^3^)
Cultivar	***	NS	*	**	**	NS	**	NS	**	NS	***	NS
WS Trt	***	***	***	***	***	NS	***	***	***	NS	NS	NS
Cul X WS Trt	*	NS	NS	NS	NS	*	NS	**	NS	NS	*	NS
Si Trt	**	NS	NS	NS	NS	NS	NS	NS	NS	NS	NS	NS
Cul X Si Trt	NS	NS	NS	NS	NS	NS	NS	**	NS	NS	NS	NS
WS Trt X Si Trt	NS	NS	NS	NS	NS	*	NS	**	NS	NS	NS	NS
Cul X WS Trt X Si Trt	**	**	NS	*	NS	NS	*	*	NS	NS	NS	NS
**Physiological traits**												
**Source of variation**	Pnet (µmol m^−2^s^−1^)		Cond (mol m^−2^ s^−1^)	C_i_/C_a_	Fv’/Fm’	ETR (µmol electron m^−2^s^−1^)	Trans (mmol m^−2^ s^−1^)		WUE (µmol CO_2_ mol^−1^ H_2_O)			
Cultivar	NS		NS	NS	*	NS	NS		NS			
WS Trt	NS		***	***	**	*	***		***			
Cul X WS Trt	NS		*	NS	NS	*	NS		NS			
Si Trt	***		**	**	***	***	***		***			
Cul X Si Trt	*		*	NS	NS	NS	NS		NS			
WS Trt X Si Trt	NS		*	***	NS	NS	***		***			
Cul X WS Trt X Si Trt	NS		NS	NS	NS	*	NS		NS			

The significance levels ***, **, *, and NS represent *p* ≤ 0.001, *p* ≤ 0.01, *p* ≤ 0.05, and *p* > 0.05, respectively. NS: non-significant. Cul: cultivar, WS Trt: water stress (limiting) treatment, Si Trt: silicate treatment. The observations were recorded at 45 days after emergence of seedling.

## Data Availability

Not applicable.

## Supplementary Materials

The following supporting information can be downloaded at: https://www.mdpi.com/article/10.3390/plants11131687/s1, Figure S1; Schematic illustration of the layout of experiments; Figure S2: Plastic shelter to protect plants from rain.
