# Supplementary material for: Silicon Enhances Plant Vegetative Growth and Soil Water Retention of Soybean (Glycine max) Plants under Water-Limiting Conditions
Source: Plants (Basel). 2022 Jun 25;11(13):1687. doi: 10.3390/plants11131687 (PMC9268825; doi:10.3390/plants11131687)
Supplement: Supplementary file 1 [file plants-11-01687-s001.zip › supplementary materials/Supplementary Figure1.pptx]

## Slide 1
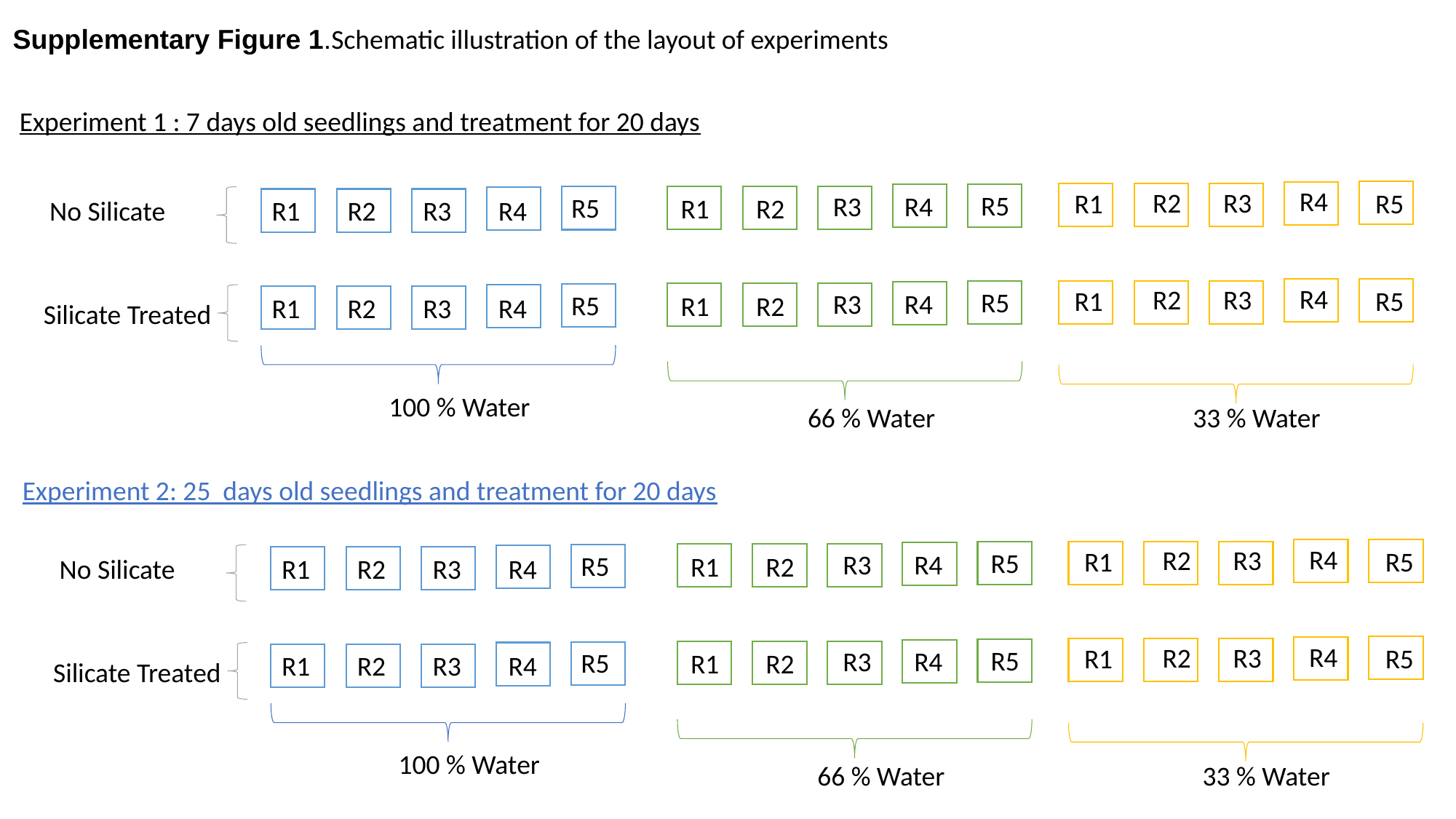

Supplementary Figure 1.Schematic illustration of the layout of experiments
Experiment 1 : 7 days old seedlings and treatment for 20 days
R4
R2
R3
R1
R5
R4
R2
R3
R1
R5
33 % Water
R5
R3
R4
R1
R2
R5
R3
R4
R1
R2
66 % Water
R5
R1
R2
R3
R4
R5
R1
R2
R3
R4
100 % Water
No Silicate
Silicate Treated
Experiment 2: 25 days old seedlings and treatment for 20 days
R4
R2
R3
R1
R5
R4
R2
R3
R1
R5
33 % Water
R5
R3
R4
R1
R2
R5
R3
R4
R1
R2
66 % Water
R5
R1
R2
R3
R4
R5
R1
R2
R3
R4
100 % Water
No Silicate
Silicate Treated
