# Supplementary figures and images for: Silicon Enhances Plant Vegetative Growth and Soil Water Retention of Soybean (Glycine max) Plants under Water-Limiting Conditions
Source: Plants (Basel). 2022 Jun 25;11(13):1687. doi: 10.3390/plants11131687 (PMC9268825; doi:10.3390/plants11131687)

## Slide 1
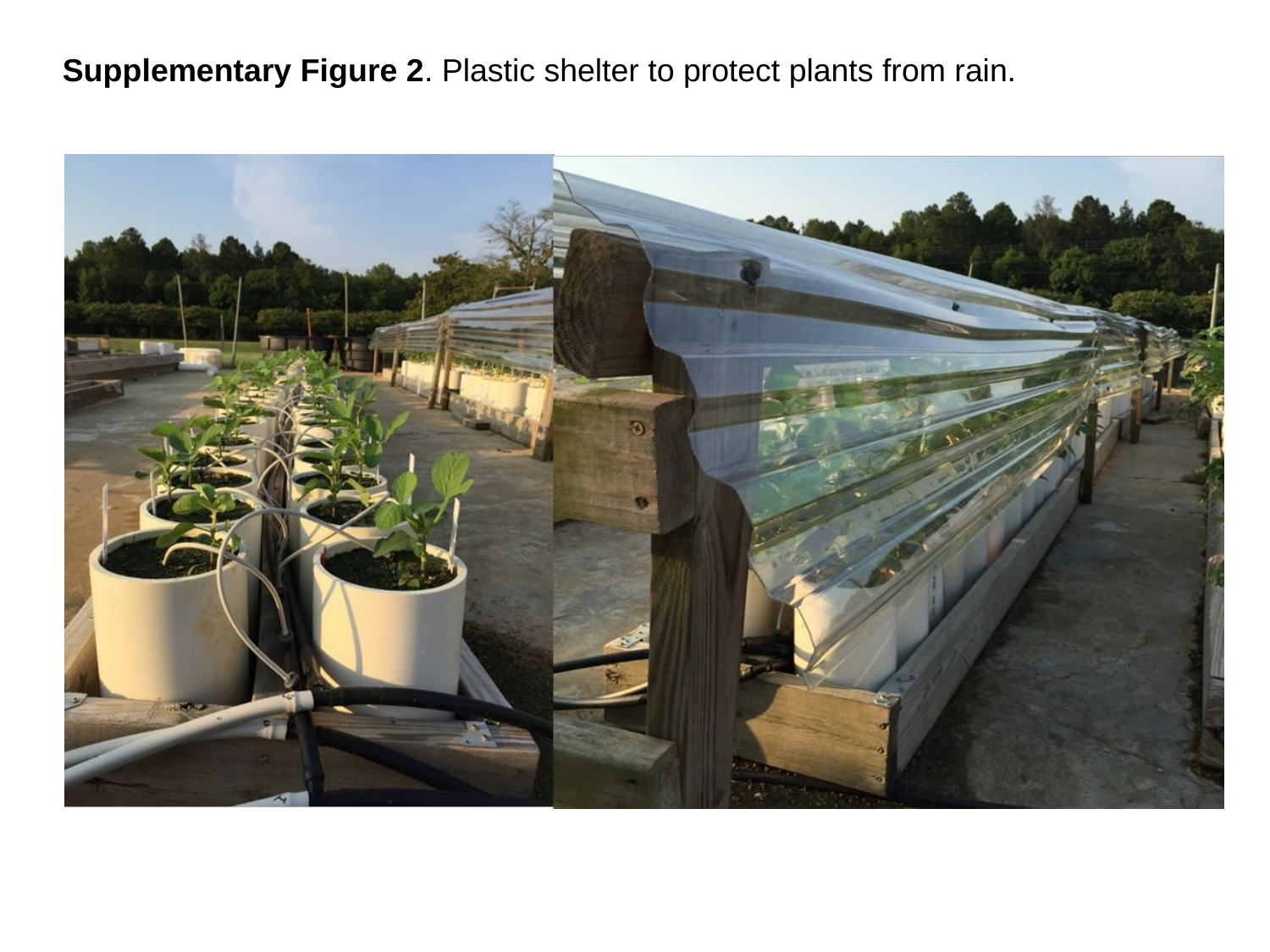

Supplementary Figure 2. Plastic shelter to protect plants from rain.

Supplement: Supplementary file 1 [file plants-11-01687-s001.zip › supplementary materials/Supplementary Figure 2.pptx]
